# Do Environmental Regulations Facilitate a Low-Carbon Transformation in China’s Resource-Based Cities?

**DOI:** 10.3390/ijerph20054502

**Published:** 2023-03-03

**Authors:** Wancheng Xie, Andrew Chapman, Taihua Yan

**Affiliations:** 1 School of Economics and Business Administration, Chongqing University, Chongqing 400030, China; 2 International Institute for Carbon Neutral Energy Research (I2CNER), Kyushu University, Fukuoka 819-0395, Japan

**Keywords:** environmental regulations, carbon emissions, carbon emission efficiency, low-carbon transformation, resource-based city

## Abstract

Resource-based cities (RBCs) are not only important for ensuring national resource and energy security, but they also face serious ecological and environmental problems. To achieve China’s carbon peaking and neutrality goals in the coming years, RBCs’ achievement of a low-carbon transformation has become increasingly significant. The core of this study is an investigation as to whether governance, including environmental regulations, can facilitate the low-carbon transformation of RBCs. Based on RBC data from 2003 to 2019, we establish a dynamic panel model to research the influence and mechanism of environmental regulations on low-carbon transformation. We found that China’s environmental regulations facilitate a low-carbon transformation in RBCs. Mechanism analysis identified that the environmental regulations facilitate the low-carbon transformation in RBCs by strengthening foreign direct investment, enhancing green technology innovation and promoting industrial structure upgrading. Heterogeneity analysis found that the environmental regulations play a greater role in facilitating the low-carbon transformation of RBCs in regions with more developed economies and less dependence on resources. Our research provides theoretical and policy implications for environmental regulations for the low-carbon transformation of RBCs in China, applicable to other resource-based areas.

## 1. Introduction

Global warming and carbon emission reduction are being focused on by scholars around the world [1,2]. Due to an extensive economic growth model, China’s carbon emissions have ranked number one in the world [3], and the total carbon emissions in 2020 were approximately 9.99 billion tons, accounting for 30.93% of global emissions [4]. Faced with increasing carbon emissions, many countries have formulated emission reduction targets [5]. Meanwhile, global economic development has suffered significantly due to the outbreak of COVID-19 and policy uncertainty. According to the World Economic Situation and Prospects (WESP; [6]), global economic growth in 2019 was the lowest in nearly a decade, slipping to just 2.3%. Further, due to the COVID-19 pandemic, the world economy shrank by 4.3% in 2020. Accordingly, to alleviate the pressure of economic deterioration and environmental pollution, the facilitation of a low-carbon transformation with the goal of achieving economic development and carbon emission reduction is essential for sustainable development and engendering a win-win outcome for low-carbon transition implementing nations.

Resource-based cities (RBCs) have made significant contributions to China’s economic growth [7]. Due to the existence of rich natural resources, the development of RBCs relies on the mining and processing of natural resources, thereby forming a typically high-carbon development model [8]. There are about 262 resource-based areas in China (about 42.7% of the total cities in China), which provide a basic guarantee for China’s resource and energy needs [9]. The low-carbon transformation of RBCs is essential for achieving the “30/60” dual carbon goal. The low-carbon transformation of RBCs is a dynamic transformation process of reducing total carbon emissions and improving carbon emission productivity levels. To comprehensively evaluate the low-carbon transformation in RBCs, we measure the twin dimensions of carbon emissions and carbon emission efficiency (CEE). However, the “resource curse” hypothesis [10] states that a richness of natural resources will hinder economic growth. Meanwhile, the “carbon curse” hypothesis [11] states that a rich endowment of natural resources will increase carbon emission intensity. Therefore, RBCs with rich natural resources may suffer from both the “resource curse” and “carbon curse,” making it more difficult to achieve a low-carbon transformation.

Environmental regulations are one way to solve environmental problems and engender emission reduction, and have been widely used in developed and developing countries [12,13]. The Chinese government has issued the Low Carbon City Pilot Policy [14], the Law on Environmental Protection [15], the Law on Prevention and Control of Air Pollution [16] and other policies to limit the carbon emissions of enterprises. This is due to the external nature of pollution reduction and the necessity for strong environmental supervision to effectively constrain the current emission behavior. However, the influence of environmental regulations on carbon emission reduction and the economic development of RBCs is complicated. Appropriate environmental protection laws and regulations will force some high-polluting enterprises to transform and carry out technological innovation to inhibit carbon dioxide emissions. In contrast, the theory of the “Green Paradox” [17] states that strict environmental supervision may accelerate the mining of fossil fuels in the short term, concurrently increasing carbon emissions. In addition, strict environmental protection laws and regulations will augment high production-cost enterprises in the short term and hinder economic growth. However, even if environmental regulations generate an innovation-compensatory effect by stimulating enterprise-level technological innovation, whether this will engender a win-win situation remains to be seen [18]. Therefore, whether environmental regulations can help resource cities avoid the “carbon curse” and achieve low-carbon transformations is still unclear.

Based on the above analysis, we propose to address the following three questions: (1) for Chinese RBCs affected by the “resource curse” and “carbon curse,” can environmental regulation effectively facilitate a low-carbon transformation of RBCs? (2) What are the pathways through which environmental regulations affect the transformation toward low-carbon status in RBCs? (3) What is the heterogeneity of environmental regulations on the low-carbon transformation of RBCs under varying resource endowment levels and in different regions? Clarifying these issues will aid with sustainable development and low-carbon transformation in China, and will also have implications for the sustainable development of other regions and nations with rich natural resource endowments.

Bearing these questions in mind, the main purpose and contribution of this study can be divided into three parts: (1) in the context of universal RBCs suffering from the “resource curse” and “carbon curse,” we will examine whether environmental regulations can effectively promote the improvement of carbon emission reduction and carbon emission efficiency, thereby facilitating a low-carbon transformation in RBCs. (2) This study will further elucidate the impact mechanism of environmental regulations on the low-carbon transformation of RBCs from three aspects: foreign direct investment (FDI), green technology innovation (GTI) and industrial structure upgrading (ISU). (3) Based on the nuances among resource-based regions in China, we investigate the influence of environmental regulations in different regions and at different resource endowment levels toward low-carbon transformation, in order to devise more appropriate policies for RBCs.

The remainder of this paper is structured as follows. Section 2 is devoted to the literature review and mechanism analysis. Methodology and data are detailed in Section 3. Empirical results are presented and discussed in Section 4. Section 5 offers conclusions. Outlines policy implications and limitations are presented in Section 6.

## 2. Literature Review and Mechanism Analysis

### 2.1. Literature Review

Since the 1760s, the outbreak of the industrial technology revolution has accelerated the exploitation and use of natural resources including coal, iron and oil, and cities and regions with natural resource mining and processing as their main industry have subsequently emerged [7]. The existing literature on RBCs has been mainly focused on aspects of the evolutionary cycle, i.e., the “resource curse” and “carbon curse” problems and transformation-related issues. With regard to theoretical analysis, Lucas [19] divided the development cycle of RBCs into four stages: construction, development, transformation and maturity. Moreover, Bradbury [20] put forward the theory that RBCs experience a period of decline and closure. The second aspect, referred to as the “resource curse” problem [10], whereby RBCs are generally unsustainable due to over-reliance on resources and inefficient use of these resources [21]. On this basis, Friedrichs and Inderwildi [11] proposed the hypothesis of the “carbon curse,” which states carbon emissions and pollution are more serious in resource-rich regions. The over-reliance on fossil fuels (e.g., coal) during the development of RBCs increased carbon emissions [22]. The third common aspect is the transformation of RBCs. RBCs often have problems such as a single industrial structure, single employment structure, high unemployment rate and low social insurance levels [23,24,25]. Meanwhile, some scholars have designed pathways for the transformation considering the aspects of industrial structure optimization, technological progress and energy efficiency [7,9,26,27]. In addition, numerous scholars have discussed RBCs from different perspectives in empirical studies, including the ecological environment, shrinking cities, industrial transformation and resource endowment [24,28,29].

As the contradiction between environmental and economic issues continues to intensify, research on carbon emissions and the efficiency of RBCs has also progressed. Cheng et al. [30] suggested that RBCs face greater pressure on carbon emission reduction due to their reliance on resources and their irrational industrial structure. Meanwhile, Hou et al. [22] found that due to the locking effect of technological innovation and human capital, the substitution of industries and technologies in RBCs requires huge investment, leading to low carbon production efficiency. Additionally, scholars have discussed the impact on CO_2_ emissions from various points of view, such as urbanization, broadband infrastructure, technological innovation, resource utilization and the green finance of RBCs [31,32,33,34]. However, research on the effect of environmental regulations on the CEE of RBCs is comparatively lacking. Only a few scholars have discussed the influence of sustainable development policies on carbon emissions in RBCs to date [35,36].

Additionally, the existing literature on environmental regulations, carbon emissions and CEE is limited and provides inconsistent results. Gao et al. [37] and Hu and Xiong [38] found that strict environmental regulations promoted the overall growth of industrial carbon productivity while Liu et al. [39] showed that environmental regulations suppress carbon emissions from local manufacturing. Some scholars postulated that environmental regulations have a positive role in their contribution toward carbon abatement [13,40]. Du and Li [41] found that environmental regulations will effectively reduce carbon emissions through pollution control. Zheng and Ge [35] found that sustainable development policies can reduce carbon emissions in RBCs. Conversely, Sinn [17] proposed the “green paradox,” indicating that the implementation of environmental policies will increase carbon emissions. Smulders et al. [42] also showed that imposing carbon tax policies can lead to such a “green paradox,” leading to increased carbon emissions in the interim. Yang et al. [43] and Albulescu et al. [44] identified that environmental regulations aggravated regional carbon emissions. Song and Han [45] also identified that environmental regulations hindered carbon production efficiency. Moreover, some scholars have also found that the influence of environmental regulations on carbon emissions displays an inverted U-shape [46,47]. Guo and Chen [48] claimed that with the shifting of the intensity of environmental regulations from weak to strong, impacts also shift away from the “green paradox” effect, toward the “reverse carbon emission reduction” effect.

To summarize this body of literature, scholars have conducted relatively rich studies on RBCs. However, for RBCs suffering from the “resource curse” and “carbon curse,” it is necessary to supplement the existing literature with research on the relationship between environmental regulations and the low-carbon transformation of RBCs. Table 1 lists a comprehensive summary of the identified literature on environmental regulations and carbon emissions.

Table 1 draws attention to the fact that there are relatively few studies on environmental regulations and low-carbon transformation, especially for RBCs. In addition, the mechanism underpinning environmental regulation’s impacts on carbon emissions and carbon emission efficiency remains unclear. Therefore, this study will be conducted cognizant of the following aspects: (1) we measure the low-carbon transformation of RBCs from the twin dimensions of carbon emissions and CEE, utilizing a generalized method of moment (GMM) approach to examine the influence of environmental regulations on the low-carbon transformation of RBCs. (2) Further, we explore the mechanism of how environmental regulations affect the low-carbon transformation in RBCs to enrich the overall analytical framework for carbon emission evaluations. (3) In order to derive the precise policy recommendations, we discuss the heterogeneity of environmental regulations on the low-carbon transformation of RBCs from two discrete aspects: region and resource endowment level.

### 2.2. Mechanism Analysis

The key to realizing the low-carbon transformation of resource-based cities is to reduce carbon emissions, an issue typically considered as a negative externality. According to the externality theory [49], government environmental regulations such as environmental taxes can solve the problem of the negative externality of environmental pollution [50]. In addition, the current thinking on the influence of environmental rules on the low-carbon transformation mainly falls under two broad views known respectively as the “promoting effect” and the “inhibiting effect.” The “promoting effect” is manifested via the setting of environmental access thresholds and carbon emission standards for major polluting industries and strictly controlling the total amount of carbon emissions allowed, before the approval of production projects, so to advance the low-carbon transformation. In addition, in order to reduce the cost of environmental governance, resource-based enterprises are required to strengthen the research of low-carbon technologies, which help improve carbon emission efficiency [51]. The “inhibiting effect” is mainly manifested as the “green paradox” and the “race to the bottom.” The “green paradox” refers to the phenomenon whereby governments introduce environmental policies (such as carbon taxes) to control climate change, which causes the price of fossil fuels to fall. If fuel owners predict that climate policies will become more stringent in the future, this may promote the sale of fossil fuels in large quantities in the short term, resulting in a substantial increase in fossil energy consumption and an increase in carbon emissions during this period [17]. In the case of the “race to the bottom,” the promotion mechanism of Chinese officials is based on GDP growth assessment, which leads to local governments’ pursuit of urban economic benefits while ignoring environmental governance, also resulting in an increase in carbon emissions [52,53]. However, with the intensification of global problems such as environmental pollution, the coordinated development of the environment and the economy has become an important part of official performance assessments. Meanwhile, the dual carbon targets proposed the Chinese government have strengthened regional environmental regulations by quantifying the emissions of major pollutants and rates of resource outputs. These measures can help limit high carbon-emitting behavior of resource enterprises, forcing them to eliminate outdated technologies, strengthen research and development and the application of low-carbon technologies, promoting the realization of the low-carbon transformation of RBCs. Therefore, the following research hypotheses are proposed:

**Hypothesis 1 (H1).**  *Environmental regulations can facilitate a low-carbon transformation in RBCs.*

Environmental regulations set the threshold for the inflow of foreign capital through market access, emission standards, etc. When environmental regulations are weak, environmental costs become as important as labor costs and can attract the inflow of FDI. However, this may lead to the transfer of technologically obsolete and polluting enterprises, which will pollute the environment of the host country [54,55]. With the gradual improvement of the environmental regulation system, the environmental treatment costs of foreign enterprises also increase, which may lead to the transfer of foreign investment to other countries with lower environmental regulations, reducing domestic FDI. However, there is also some evidence that the strengthening of environmental regulations leads to the improvement of national institutional quality to some extent, and that FDI tends to flow into countries with better institutional quality [56]. Therefore, the impact of the improvement of environmental regulations on FDI is uncertain. Additionally, strict environmental regulations will curb FDI with “high energy consumption and high emissions” and attract foreign investment enterprises with “clean, green and low carbon” qualities [57]. The “pollution halo” effect suggests that foreign-funded enterprises with advanced technology can promote local resource and energy utilization efficiency and the low-carbon transformation through financing and technology diffusion [58]. Next, we propose to evaluate the following hypothesis:

**Hypothesis 2 (H2).**  *Environmental regulations have an indirect influence on the low-carbon transformation of RBCs by influencing FDI.*

Environmental regulations can affect GTI through the “compliance cost” effect and “innovation compensation” effect [59], thus indirectly facilitating the low-carbon transformation of RBCs. The “compliance cost” effect states that enterprises in response to environmental standards and due to the increased environmental costs of enterprises will crowd out R&D investment, which may hinder the progress of green technology [60]. However, the Porter hypothesis [61] states that appropriate environmental regulations can encourage enterprises to increase research funds and improve the enterprises’ GTI. The “innovation compensation” effect can not only partially or completely offset the additional costs incurred by enterprises in complying with environmental regulations, but also enables enterprises to gain a market-competitive advantage [62]. In addition, GTI can facilitate the low-carbon transformation of RBCs through energy-saving innovations in the pollution generation stage, terminal treatment of pollution emissions and improvement of resource and energy utilization efficiency [63]. Driven by “carbon emission reduction targets,” the government has generally adopted continuous and rigorous environmental regulations. A reasonable response plan for enterprises is more likely to enhance investment in GTI R&D to meet the challenges brought about by environmental regulations toward their long-term development [64], which is beneficial for the low-carbon transformation of RBCs. Further, this paper proposes the following hypothesis:

**Hypothesis 3 (H3).**  *Environmental regulations have an indirect impact on the low-carbon transformation of RBCs via their influence on GTI.*

Environmental regulations can affect the upgrading of industrial structure through the survival of the fittest and industrial transfer effects, thus indirectly affecting the low-carbon transformation of RBCs. The improvement of environmental regulation intensity will raise the environmental access threshold and increase the production cost of enterprises, thus eliminating small-scale enterprises, those with low production efficiency and high emission levels, while high value-added industries, clean industries and technology-intensive industries will gain advantages via the survival of the fittest mechanism [65]. Meanwhile, increased environmental regulations will force some high-emission industries to transfer to other regions and countries with weaker emission regulations [66], thereby achieving industrial structure transformation and reducing carbon emissions. The ISU of RBCs depends on the regulations and guidance provided by government industrial policies. By establishing a negative list management mode (whereby the government stipulates that enterprises are prohibited to invest in certain economic sectors and industries in the region) of environmental access, the government can prohibit investment in high-emission, high energy use and highly polluting industries, guide the entry of high-tech, clean industries and facilitate the optimization of the overall industrial structure. In addition, industrial structure adjustment can improve resource utilization and carbon emission reductions [64], promoting the low-carbon transformation of RBCs. Finally, this paper proposes the following hypothesis:

**Hypothesis 4 (H4).**  *Environmental regulations have an indirect impact on the low-carbon transformation of RBCs via their influence of ISU.*

**Figure 1:**
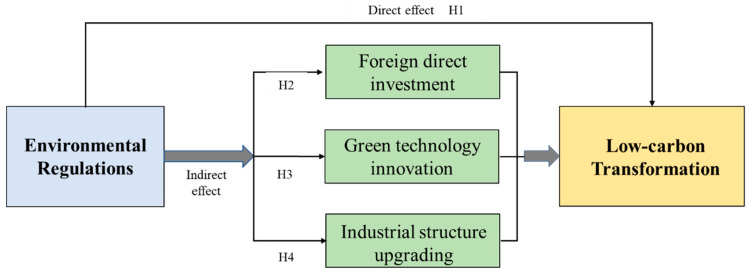
The mechanism of environmental regulations on low-carbon transformation in RBCs.

Through mechanism analysis, this paper analyzes and clarifies the mechanism relationship of environmental regulations on the low-carbon transformation in RBCs, as shown in Figure 1:

## 3. Methodology and Data

### 3.1. Methodology

To evaluate the causal effect between environmental regulations and the low-carbon transformation of RBCs, we established the following model based on the methods of Gao et al. [37], Hu and Xiong [38] and Wang and Zhang [47]:

(1) $$LOC_{it}=\alpha_{0}+\alpha_{1}ER_{it}+\alpha_{2}X_{it}+\gamma_{i}+\delta_{t}+\varepsilon_{it}$$

In Equation (1), $LOC_{it}$ is the explained variable, which denotes the low-carbon transformation of RBCs, including carbon emissions (CE) and carbon emission efficiency (CEE). $ER_{it}$ is the explanatory variable, which represents environmental regulations. $X_{it}$ represents the matrix of control variables, including per-region capita GDP (PGDP), resource endowment (RE), marketization level (MAR), government intervention (GI) and financial development (FD). i and t represent the city and year, respectively. Because the environmental regulations vary greatly between the different regions, this study controls for the city and time-fixed effects, namely $\gamma_{i}$ and $\delta_{t}$, respectively, while $\varepsilon_{it}$ denotes a disturbance term.

Autoregressive and interactive effects may exist between explanatory variables and explained variables. Therefore, there may also be bidirectional causality between environmental regulations and the low-carbon transformation, leading to endogeneity problems. To alleviate the endogeneity problem, Arellano and Bond [67] and Blundell and Bond [68] proposed the generalized moment estimation method (GMM) by selecting high-order lagged variables as instrumental variables. The existing research on the estimation methods of GMM is mainly divided into system GMM (S-GMM) and difference GMM (D-GMM). Compared with the D-GMM method, the S-GMM method effectively solves the problem of weak tool variables and the endogenous problem in the model. Therefore, leveraging Equation (1), this paper introduces the one-period-lagged term of the explained variable and uses the S-GMM method for empirical analysis. The specific dynamic panel regression model in this paper is given in Equation (2):

(2) $$LOC_{it}=\alpha_{0}+\alpha_{1}LOC_{i,t-1}+\alpha_{2}ER_{it}+\alpha_{3}X_{it}+\gamma_{i}+\delta_{t}+\varepsilon_{it}$$

Additionally, environmental regulations can indirectly affect the low-carbon transformation of RBCs through the transmission channels of FDI, GTI and ISU. We used the mediating effect model [69] to test how environmental regulations affect the transmission mechanism of low-carbon transformations (Equations (3) and (4)):

(3) $$M_{it}=\beta_{0}+\beta_{1}M_{i,t-1}+\beta_{2}ER_{it}+\beta_{3}X_{it}+\gamma_{i}+\delta_{t}+\varepsilon_{it}$$

(4) $$LOC_{it}=\delta_{0}+\delta_{1}LOC_{i,t-1}+\delta_{2}ER_{it}+\delta_{3}M_{it}+\delta_{4}X_{it}+\gamma_{i}+\delta_{t}+\varepsilon_{it}$$

where $M_{it}$ denotes a set of mediating variables, including FDI, GTI and ISU. The other settings are the same as for Equation (2). Equation (3) represents the influence of environmental regulations on mediating variables and Equation (4) mainly analyzes the combined influence of environmental regulations and mediating variables on the low-carbon transformation of RBCs.

### 3.2. Variables and Data

#### 3.2.1. Explained Variable

To comprehensively evaluate the low-carbon transformation of RBCs, we measured the two dimensions of carbon emissions (CE) and carbon emission efficiency (CEE).

For carbon emissions (CE), based on the methods of Oda et al. [70], we aggregated and collected the raster data of CO_2_ emissions from the Open-Data Inventory for Anthropogenic Carbon dioxide (ODIAC), to obtain carbon emissions data for resources-based cities. Based on space-based nighttime light data and power plant locations, ODIAC provides global carbon dioxide emissions images at a resolution of 1 × 1 km, providing highly accurate urban carbon emissions data. ODIAC carbon emissions data has been previously applied to the evaluation of urban emissions estimation and carbon cycles [71]. Meanwhile, to eliminate the interference of heteroscedasticity, we took the logarithm of the carbon emissions to represent the CE.

Carbon emission efficiency (CEE) refers to the maximum economic output and the minimum CO_2_ emissions that can be obtained under a variety of input factors such as capital, labor and energy. The existing research adopted the data-enveloping analysis (DEA) model to measure the CEE [72,73]. The DEA method can incorporate the inputs, desirable outputs and undesirable outputs into the same efficiency evaluation system, thereby achieving the measurement of urban carbon emissions efficiency [74]. In addition, to eliminate the “technical regress” of the traditional DEA method, this paper refers to the method of Shestalova [75] and uses an output-oriented sequential DEA method to calculate the CEE. We choose capital (K), labor (L) and energy (E) values of RBCs as input indicators, GDP as the desirable output indicator and carbon emissions as the undesirable output indicator. Referring to the method of Ke and Xiang [76], capital (K) input is represented by the capital stock of RBCs. Using 2003 as the benchmark year, we used the sustainable trading method to estimate the capital stock of RBCs to represent capital investment indicators. Labor (L) input is represented by the total number of employees in RBCs. Energy (E) input uses electricity consumption as an indirect proxy due to the highly positive correlation between energy consumption and electricity consumption [77]. The desirable output is expressed by using the actual regional GDP of RBCs. In addition, carbon emissions were chosen as a proxy of the undesirable output.

#### 3.2.2. Explanatory Variable

Environmental regulations (ER) are mainly implemented to protect the environment for the purpose of the regulation of various pollutants. The measurement methods for environmental regulations in the literature are as follows. Some scholars use a single index for measurement, such as the number of policies and regulations promulgated, investment in environmental pollution control and the collection of sewage charges etc. [78,79]. Other scholars have used alternative indicators for measurement, such as total energy consumption per unit of GDP and per-capita income [80]. In addition, some scholars measured impacts through a comprehensive index method, such as the comprehensive calculation of the removal rate of major pollutants utilizing the sensitivity analysis method to measure environmental regulation [81,82,83,84]. Considering the research topic of this study, the above environmental regulation indicators are strongly correlated with low-carbon transition developments, which has obvious endogeneity problems. In addition, since environmental regulations take various forms such as administrative orders and economic constraints, the above indicators may not be appropriate to reflect comprehensive government environmental regulation policies. Therefore, we draw on the method of Chen et al. [85] and manually sort regulatory documents issued by various RBCs from 2003 to 2019 from the PKULAW database with the keywords of “environmental protection, emission reduction, low-carbon” etc. There were 1956 local laws and regulations, 560 local government regulations, 23,508 local normative documents and 47,925 local working documents considered in total. Specifically, we select the number of these government regulation documents as a proxy index to measure environmental regulations in RBCs, and take the logarithm of these values.

#### 3.2.3. Mechanism Variables

First, we consider FDI. The relatively loose environmental regulations in resource-based cities are conducive to the entry of foreign investment [86]. Meanwhile, the advanced technology and equipment brought by environmentally friendly foreign investment enterprises are conducive to the ecological improvement and low-carbon transformation of RBCs [87]. Therefore, to explore whether FDI is a transmission channel for environmental regulations to enhance the low-carbon transformation of RBCs, we use the amount of foreign capital utilized to represent FDI, logarithmically.

Next, we consider GTI. GTI is an intrinsic factor to achieve the low-carbon transformation of RBCs. To summarize the existing literature, there are three main methods for GTI measurement. First, from the perspective of innovation input, R&D expenditure is generally used as a substitute variable for GTI [88]. Second, methods exist which construct comprehensive indicators of input and output [89]. In addition, some scholars measure GTI from the viewpoint of technological innovation output, including the number of green invention patents and green patent applications based on the “IPC Green Inventory” maintained by WIPO [90]. Many studies have also shown that patents filed in green technologies are a proxy indicator for approximating GTI [51]. Therefore, referring to the method of Lai et al. [91], GTI in RBCs in this study is represented by the natural logarithm of the number of green patent applications plus 1.

Finally, we consider ISU. Environmental regulation can affect the ISU of RBCs through the “survival of the fittest” mechanism. In addition, compared with secondary industries, tertiary industry has lower comparative energy consumption and pollutant emissions, especially for RBCs dominated by natural resource development. The optimization and upgrading of industrial structure is an important driving force for the low-carbon transformation of RBCs. Therefore, to explore whether the upgrading of industrial structure is a transmission channel for environmental regulations to enhance the low-carbon transformation of RBCs, we select the ratio of the output value of tertiary industry to secondary industry to represent the upgrading of the industrial structure of RBCs.

#### 3.2.4. Control Variables

As a control variable, we consider the regional economic development level (PGDP): the level of the economy has a significant impact on the low-carbon transformation of RBCs. We use the logarithm of local per-capita GDP as a measure of the economic development level of RBCs. Next, we consider marketization level (MAR): the improvement of marketization facilitates the rational utilization of resources, which promotes the low-carbon transformation of RBCs. We use the proportion of urban self-employed and private economy employees in total employment to express this variable. Additionally, we consider resource endowments (RE), where areas with sufficient resource endowments in RBCs may fall into the trap of the “resource curse,” which affects both innovation and the low-carbon transformation [92]. We use the ratio of total employment in extractive industries within total employment to represent this variable. In addition, we consider government intervention (GI). The financial intervention of local governments can have a significant impact on the local economy and environment. We use the ratio of expenditure in the general local budget to regional GDP to express this variable. Finally, we consider financial development (FD), represented by the ratio of financial institutions’ loan balance to regional GDP. The main variables considered in this study are defined in Table 2.

#### 3.2.5. Data Sources

This paper takes a total of 1938 observed variables from 114 RBCs in China from 2003 to 2019 as the research object. The data of relevant variables are extracted from the China City Statistical Yearbook, the China Environment Statistical Yearbook [93], the Express Professional Superior [94] database, Peking University Fabao Database [95] and ODIAC database. Missing values are interpolated. The statistical description of the main variables is shown in Table 3.

## 4. Empirical Results and Discussion

### 4.1. Baseline Regression Results

The system generalized moment estimation (S-GMM) method is widely applied in the measurement of dynamic panel models [46,47]. According to precedential model construction, we use the S-GMM method to empirically test the impact of environmental regulations on the low-carbon transformation of RBCs. Moreover, Arellano-Bond (AR) and Hansen tests were carried out to ensure the credibility of the models and the validity of instrumental variables. Additionally, to increase the reliability and feasibility of the model, columns 1 and 2 represent the models without control variables, and columns 3 and 4 represent models with control variables. Results are detailed in Table 4.

We found that the *p*-value of AR(1) is less than 0.1 and the *p*-value of AR(2) is greater than 0.1 in all model results, indicating that there is first-order serial correlation in the residuals of all model results, but no second-order serial correlation. Thus, the estimators of the model are consistent. Meanwhile, the Hansen test values of all regression results are greater than 0.15, indicating that the instrumental variables used in the model are plausible. We can see from column 1 to column 4 that in the models with and without control variables, the regression coefficient of ER and CE is negatively correlated at the 1% significance level and positively correlated with CEE at the 1% significance level, indicating that the control variables do not change the regression results. It can be identified from column 3 that the regression coefficient between ER and CE is −0.014. Specifically, on average, a 1% increase in the number of government documents related to environmental regulations reduces carbon emissions of RBCs by 0.014%. In addition, it can be interpreted from column 4 that the regression coefficient between ER and CEE is 0.006. Specifically, on average, a 1% increase in the number of government documents related to environmental regulations increases the carbon efficiency of RBCs by 0.006 units. The results indicate that environmental regulations can effectively improve the low-carbon transformation of RBCs from two dimensions: reducing carbon emissions and improving the CEE of RBCs, verifying hypothesis H1. Environmental regulations force highly polluting enterprises to reduce carbon emissions by charging sewage fees and environmental taxes, reducing carbon emissions in RBCs. In addition, reasonable selection and formulation of environmental regulations can enhance the improvement of resource and energy utilization efficiency, promoting the CEE of RBCs.

Considering the regression results of other control variables, the coefficients of RE in columns 3 and 4 are significantly positive and negative, which means that resource endowment impedes the reduction of carbon emissions and the improvement of CEE in RBCs respectively. This finding confirms the existence of the resource curse and carbon curse effects in RBCs [10,11]. Next, the regression coefficient between PGDP and CE is not significant, but the regression coefficient between PGDP and CEE is significantly positive. Regions with a higher economic development level can attract more capital and high-level talent to promote advanced low-carbon and clean technologies, which is conducive to CEE improvement in RBCs. Additionally, the regression coefficients of MAR with both CE and CEE are not significant, indicating that the marketization level at the present stage has no noticeable impact on the low-carbon transformation of RBCs. In addition, the coefficient of GI and CEE is significantly negative, revealing that excessive government intervention inhibits the low-carbon transformation of RBCs [96]. Finally, the coefficient of FD and CE is significantly negative, while that of CEE is significantly positive. This identifies that financial development can decrease carbon emissions and facilitate CEE of RBCs [97].

### 4.2. Mechanism Analysis

We employ the mediation effect model to analyze the mechanisms of environmental regulations affecting the low-carbon transformation in RBCs from three aspects: FDI, GTI and ISU.

#### 4.2.1. The Intermediary Role of FDI Channels

Regression results of our mechanism analysis for FDI are shown in Table 5.

As we observe from column 1 of Table 5, the coefficient between ER and FDI is significantly positive, which means that ER can increase FDI in RBCs. Meanwhile, as can be seen from the total effect outcomes in column 2 and 3, the coefficient between FDI and CE is significantly negative, which means that FDI can reduce carbon emissions in RBCs. The regression results show that FDI plays an intermediary role between environmental regulations and carbon emissions of RBCs. On the other hand, the coefficient between FDI and CEE is significantly positive, indicating that FDI can improve CEE of RBCs. The regression results show that FDI plays an intermediary role between environmental regulations and CEE of RBCs. This research conclusion is consistent with Yang et al. [43], suggesting that environmental regulations can indirectly reduce carbon emissions by promoting FDI. The implementation of strict environmental regulations will reduce the profits of polluting foreign enterprises and hinder the inflow of polluting foreign investment [98]. In addition, the rational implementation of environmental regulations can attract environmentally friendly foreign enterprises [99], which can facilitate the low-carbon transformation by introducing advanced low-carbon technology and management experience. Especially in response to serious environmental pollution in RBCs, the government has issued a series of strict environmental regulations, which can effectively raise the threshold for investment and facilitate the entry of environmentally friendly foreign investment into RBCs. As a result, the diffusion of low-carbon and clean production technology in RBCs is promoted, thereby facilitating the low-carbon transformation of RBCs.

#### 4.2.2. The Intermediary Role of GTI Channels

Regression results of mechanism analysis for GTI are shown in Table 6.

From column 1 of Table 6, the coefficient between ER and GTI is significantly positive, and the coefficient between GTI and CE in column 2 is significantly negative. This indicates that environmental regulations can indirectly reduce the carbon emissions of RBCs through the intermediary channel of GTI. In addition, the coefficient between GTI and CEE is significantly positive, revealing that GTI can improve the CEE of RBCs. This indicates that environmental regulation can indirectly promote the CEE of RBCs through the intermediary channel of GTI. In addition, Yang and Yang [89] found that appropriate environmental regulatory measures and policies can effectively promote urban GTI in the context of China’s green development. Appropriate environmental regulations can promote enterprises to R&D investment through the “innovation compensation” effect, which is conducive to GTI in RBCs. Environmental regulations establish certain environmental standards and requirements, and impose mandatory constraints on resource-based enterprises. To avoid punishment, enterprises need to adopt more environmentally friendly production methods and technologies, which promotes technological innovation and green technology application. Additionally, GTI can effectively facilitate improved utilization efficiency of resources and energy [100,101], which is conducive to the low-carbon transformation of RBCs [102].

#### 4.2.3. The Intermediary Role of ISU Channels

Regression results of mechanism analysis for ISU are shown in Table 7.

As can be seen from column 1 of Table 7, the coefficient between ER and ISU is significantly positive, and the coefficient between ISU and CE in column 2 is significantly negative. The regression results show that environmental regulations can indirectly impact the carbon emissions of RBCs by promoting the upgrading of the industrial structure. Since the secondary industry is the main carbon-emitting industry, the upgrading of industrial structure reduces the overall proportion of the secondary industry and increases the proportion of the tertiary industry [64], thereby effectively reducing the carbon emissions of RBCs. Additionally, as can be seen from column 3, the coefficient between ISU and CEE is positive but not significant, indicating that the upgrading of industrial structure has not promoted CEE improvement in RBCs. Since the economic development of RBCs mainly depends on the secondary industry of resource exploitation [92], the upgrading of the industrial structure may have hindered economic development in the short term. Therefore, the upgrading of the industrial structure does not facilitate the CEE of RBCs. Overall, the implementation of environmental regulations can facilitate the traditional resource-based industries with high energy consumption, high emissions and low added value to gradually withdraw from the market, while promoting emerging industries with low energy consumption, low emissions and high added value to develop vigorously in RBCs. In addition, the upgrading of industrial structure can facilitate the low-carbon transformation of resource-based cities by promoting carbon emission reduction.

### 4.3. Robustness Test

To ensure credibility and reliability, we conducted a series of robustness tests, with results shown in Table 8.

First, in order to eliminate the interference of outliers and maintain the integrity of the sample information, this study undertakes a 1% data smoothing (Winsor) on all variables before re-testing. The test results are shown in columns 1–2 of Table 8, environmental regulations still decrease carbon emissions and facilitate CEE of RBCs at the 1% statistical significance level. The test results indicate the core conclusion is not disturbed by outliers, verifying the robustness of the benchmark regression results.

Secondly, stricter environmental regulations and policies have been implemented for the “2 + 26” cities in China with more serious environmental pollution [103]; these cities include 11 RBCs, whose environmental supervision policies are significantly different from other RBCs. To exclude the influence of extremely strict environmental policies on the overall research results, it is necessary to test whether the regression results are robust after removing these 11 RBCs. Columns 3–4 of Table 8 show the estimated results after removing 11 RBCs with strict environmental regulations. The sign of the regression coefficient of the main variable is consistent with the basic regression results at the 1% significance level.

Additionally, to eliminate bias caused by the lag effect, we added the first-order lag term of the explanatory variable to re-estimate the basic regression. The test results are shown in columns 5–6 of Table 8, the regression coefficients of L.ER with CE and CEE are both not significant. Meanwhile, the influence of environmental regulations on carbon emissions and CEE is still significant. The stability of the benchmark regression results is further demonstrated.

Finally, we replace explanatory variables and explore the credibility of the benchmark results. Because the intensity of environmental regulation is highly correlated with the discharge and utilization rate of major pollutants in urban industry, based on the research method of Du et al. [81] and Tian and Feng [104], we select five indicators, namely industrial wastewater discharge, industrial sulfur dioxide discharge, industrial soot (dust) discharge and the ratio of industrial solid wastes comprehensively utilized, and use the entropy method to comprehensively evaluate the implementation of environmental regulations (R-ER) in RBCs. The test results are shown in columns 7–8 of Table 8. At the 1% significance level, the coefficient of the core explanatory variable on carbon emissions is still negative, while the coefficient of the core explanatory variable on CEE is still positive. It shows that the core conclusion does not substantially change due to a change in environmental regulation measurement indicators. Overall, stability tests suggest that this paper’s empirical results are reliable.

### 4.4. Heterogeneity Analysis

According to Sustainable Development Plan for National Resource-based Cities [105] issued by the State Council of China, the state divides RBCs into growth-type, maturity-type, recession-type and regeneration-type based on the resource endowment level and economic development. Based on these divisions, we explore the heterogeneity of environmental regulations on the low-carbon transformation according to the different classifications of RBCs.

The empirical test results are shown in columns 1–8 of Table 9.

In terms of the influence between the environmental regulations and carbon emissions, the coefficient between ER and CE is negative in different types of RBCs. Except for recession-type RBCs, all the other types satisfied the 10% significance level test. In addition, the environmental regulation impacts in descending order of strength are: growth-type, maturity-type, regeneration-type and recession-type RBCs. In terms of the influence between the environmental regulations and CEE, only maturity-type and regeneration-type RBCs have a significantly positive coefficient between ER and CEE, while growth types and recession types have no significant influence. Meanwhile, we observe that environmental regulations have the strongest promoting effect on CEE of regeneration-type RBCs, with a descending order of strength: regeneration-type, growth-type, maturity-type and recession-type RBCs. Because the recession-type RBCs are faced with issues of resource exhaustion, backward economic development and low efficiency of resource utilization, environmental regulation fails to facilitate carbon emission reduction, while mature RBCs in the stable development stage with a higher level of economic and social development pay more attention to environmental problems. Meanwhile, the regeneration-type RBCs seek to eliminate dependence on resources and are pilot areas for the transformation of the economic development mode of RBCs. Therefore, environmental regulations play a stronger role in facilitating the low-carbon transformation for maturity-type RBCs with higher economic development and regeneration-type RBCs with lower resource dependence.

RBCs in Northeast China are faced with the multiple challenges of resource exhaustion, low levels of economic development and serious environmental pollution [28,106]. Meanwhile, since the state implemented the strategy of revitalizing old industrial bases such as the Northeast in 2003, the government has introduced a series of tax incentives, special investment, transfer payments and other policies to promote urban economic development. Therefore, we investigate whether the effect of environmental regulations on the low-carbon transformation of RBCs in the northeast region and non-northeast region is heterogeneous.

The estimated results are shown in columns 1–4 of Table 10.

Columns 1 and 2 show the regression results for the non-northeastern regions, where the coefficients of ER and CE are significantly negative, and the coefficients of ER and CEE are significantly positive. This shows that environmental regulations can facilitate a low-carbon transformation of RBCs in Northeast China. However, the coefficient of ER and CE is still negative but insignificant in Northeast China, as shown in column 3. In addition, the coefficient between ER and CEE becomes negative but insignificant as shown in column 4. This indicates that environmental regulations in Northeast China have failed to facilitate carbon emission reduction and CEE, and may even have had a negative effect. On the one hand, RBCs in Northeast China are characterized by a single economic structure and dual management systems [23], which leads to the failure of environmental regulations to facilitate carbon emission reduction of RBCs in Northeast China. On the other hand, although the Northeast region’s economy has developed rapidly since the launch and implementation of the old industrial base revitalization strategy, environmental governance issues have been ignored, resulting in the failure of environmental regulations to enhance the improvement of CEE. Therefore, the environmental regulations play a stronger role in facilitating the low-carbon transformation of maturity-type RBCs with lower resource dependence and regeneration-type RBCs with higher economic development.

## 5. Conclusions

According to the analysis of panel data from 114 RBCs from 2003 to 2019, this study applied the S-GMM model to examine the influence of environmental regulations on the low-carbon transformation of RBCs in China.

Based on our systematic research approach, important conclusions are threefold: first, environmental regulations can promote carbon emission reduction and the improvement of CEE to achieve the low-carbon transformation in RBCs, verifying hypothesis H1. Specifically, on average, a 1% increase in the number of government documents related to environmental regulations reduces the carbon emissions of RBCs by 0.014% and increases CEE by 0.006 units. Second, the mechanism analysis identified that environmental regulations can facilitate a low-carbon transformation of RBCs through strengthening FDI, enhancing GTI and promoting ISU, thus verifying hypotheses H2, H3 and H4. The intermediary path of FDI and GTI can jointly enhance the low-carbon transformation of RBCs through two dimensions: reducing carbon emissions and facilitating CEE. However, the intermediary path of ISU can only effectively facilitate carbon emission reduction in RBCs. Finally, heterogeneity analysis found that environmental regulations play a greater role in facilitating the low-carbon transformation of RBCs in regions with more developed economies and less dependence on resources. In addition, environmental regulations had a more significant role in facilitating the low-carbon transformation of RBCs in non-northeast regions.

## 6. Policy Implications and Limitations

Based on these findings, the following policy recommendations are proposed to facilitate a low-carbon transformation among a variety of RBCs and resource-based regions.

First, as an important pillar to reduce urban carbon emissions, policy makers should continuously improve the environmental regulations of RBCs. The central government needs to set appropriate environmental quality goals through an evaluation of the environmental quality of each RBC, encouraging local governments to actively solve problems related to environmental pollution. In addition, the government should strictly enforce responsibility for environmental remediation, ecological construction and resource compensation of resource development enterprises, and enhance the efficiency of resource utilization by guiding the intensive development and large-scale production of resources, so as to reduce carbon emissions and improve the economic benefits arising from RBCs.

Second, the government should increase the opening up and cooperation of RBCs, both internally and externally. The local governments should optimize the business environment, absorb environmentally friendly foreign enterprises, as well as introducing advanced green technologies, equipment, management concepts and high value-added project investment from developed countries and regions, so as to improve the low-carbon transformation of RBCs. In addition, policy makers should construct an appropriate environmental regulation system to improve the environmental access threshold of FDI and restrict the entry of foreign enterprises with high pollution, high emissions and high energy consumption.

Third, the government should view the transformation and upgrading of economic structures as a priority toward accelerating the low-carbon transformation of RBCs. On the one hand, the government needs to plan for industry to extend down and lengthen the supply chain, improve the efficiency of resource output utilization and develop an integrated system for the integration of petroleum refining, the chemical industry and the integration of coal and electricity. Through the planning, design and implementation of middle and downstream systems of the industrial supply chain, the aim should be to maximize economic, social and environmental benefits of enterprises, and finally achieve a green, low-carbon transformation. On the other hand, policy makers cannot achieve all their goals by blindly pursuing the upgrading of industrial structure of RBCs, but should also pay attention to the coupling and coordinated development of different industries in the process of industrial structure adjustment.

Fourth, the government should actively promote the level of green innovation in RBCs, advancing the achievement of low-carbon transformation. The government should increase financial support for enterprises’ green innovation by means of green innovation subsidies, tax cuts and lowering the financing threshold, so as to relieve the pressure of high cost and high risk of enterprises’ green and low-carbon innovation and improve the initiative and enthusiasm of the enterprises. In addition, the government needs to focus on the cultivation of green technology talent, increasing salary incentives for green technology R&D personnel, and guide universities and research institutions to advance green innovation in the fields of resource mining and utilization.

Finally, different types and regions of RBCs should implement differentiated environmental regulation policies. The government should focus on optimizing environmental regulations in declining and growing RBCs, while increasing investment and subsidies in environmental governance, so as to encourage the low-carbon transformation role of environmental regulations. Meanwhile, policy makers can enhance the environmental regulations of maturity-type and regeneration-type RBCs to encourage carbon emission reduction. For RBCs in Northeast China with serious economic and environmental problems, policymakers should increase efforts in infrastructure construction, GTI and talent introduction while constantly improving environmental regulatory policies.

This paper has some limitations, which may provide a direction for future research. The measurement of environmental regulation is relatively simple, able to be classified into command-type and market-type measurements. In the future, the impacts of different types of environmental regulations on the low-carbon transformation of RBCs could be further explored. In addition, in the case of heterogeneity analysis, future research could classify and discuss the low-carbon transformation of RBCs according to the resource attributes (such the existence of oil, coal, forests, etc.).

## Figures and Tables

**Table 1:** Environmental regulations and carbon emissions.

**Authors**	**Objective**	**Time**	**Methods**	Main Conclusions
Pei et al. (2019) [40]	Energy-intensive industries in China	2005–2015	Panel OLS	Environmental regulations directly inhibit CO_2_ emissions
Gao et al. (2019) [37]	21 major industrial sectors in China	2004–2014	Panel OLS	Environmental regulations promoted the overall growth of industrial carbon productivity
Hu and Xiong (2021) [38]	Chinese 36 industrial sectors	2000–2016	GMM	Environmental regulations facilitate carbon productivity
Ulucak et al. (2020) [13]	BRICS countries	1995–2016	Modified panel OLS	Environmental regulations promoted carbon abatement
Du and Li (2020) [41]	Chinese industrial enterprises	2000–2012	Panel fixed effect model	Environmental regulations can facilitate carbon emission reductions
Liu et al. (2021) [39]	Manufacturing panel data in China	2007–2019	The spatial Durbin	Environmental regulations suppress carbon emissions from local manufacturing
Zheng and Ge (2022) [35]	Resource-based cities	2013–2020	DID	Sustainable development policy inhibits carbon emissions in RBCs
Yang et al. (2020) [43]	30 Chinese provinces	2003–2017	The spatial econometric	Environmental regulations aggravated regional carbon emissions
Song and Han (2022) [45]	30 Chinese provinces	2006–2018	The two-tier stochastic frontier model	Environmental regulations hindered the improvement of carbon productivity
Albulescu et al. (2022) [44]	OECD countries	1990–2015	Quantile fixed-effect panel model	Environmental regulations hinder carbon reduction
Guo and Chen (2018) [48]	30 Chinese provinces	2004–2015	GMM	An inverted U-shaped curve relationship
Zhang et al. (2020) [46]	30 Chinese provinces	2008–2016	The threshold regression	Environmental regulations have a threshold effect on carbon emissions
Wang and Zhang (2022) [47]	282 Chinese cities	2003–2016	The system-generalized GMM	An inverted U-shaped relationship

**Table 2:** Variable definitions.

Variable Classification	Variable Symbol	Variable Definitions
Dependent variable	CE	The logarithm of carbon emissions
	CEE	Carbon emission efficiency calculated by the DEA
Independent variable	ER	The number of environmental laws and regulations issued by resource-based cities, using the natural logarithm
Mediating variables	FDI	The logarithm of the amount of foreign capital actually utilized
	GTI	The green patent application count plus 1, logarithmically
	ISU	Ratio of the output value of the tertiary industry to the secondary industry
Control variables	PGDP	Region per-capita GDP, taking the natural logarithm
	MAR	The proportion of urban individual and private economic employees in the total employment base
	RE	Ratio of the number of people in the extractive industry within the total number of employees
	GI	Ratio of public finance expenditure to regional GDP
	FD	Ratio of the loan balance of financial institutions to regional GDP

**Table 3:** Descriptive statistics of the variables.

Classification	Variable	N	Mean	St. Dev	Max	Min
Explained variable	CE	1938	16.570	0.808	18.553	13.939
	CEE		0.748	0.148	1.000	0.520
Explanatory variable	ER		2.901	1.112	6.477	0.000
Mediating variables	FDI		8.849	1.761	12.592	1.099
	GTI		2.341	0.874	6.960	0.000
	ISU		0.809	0.329	3.758	0.094
Control variables	PGDP		10.095	0.606	12.456	4.595
	MAR		0.116	0.121	0.581	0.000
	RE		0.933	0.306	17.141	0.014
	GI		0.185	0.050	1.027	0.031
	FD		0.737	0.268	9.622	0.033

**Table 4:** Baseline regression.

Variables	1	2	3	4
	CE	CEE	CE	CEE
L.CE	0.966 ***		0.947 ***	
	(0.008)		(0.014)	
L.CEE		0.745 ***		0.739 ***
		(0.039)		(0.026)
ER	−0.021 ***	0.006 ***	−0.014 ***	0.006 ***
	(0.002)	(0.001)	(0.003)	(0.001)
PGDP			−0.001	0.025 ***
			(0.011)	(0.006)
MAR			0.003	0.001
			(0.004)	(0.003)
RE			0.463 ***	−0.147 ***
			(0.112)	(0.026)
GI			0.049	−0.118 **
			(0.079)	(0.027)
FD			−0.041 *	0.038 ***
			(0.023)	(0.003)
Constant	0.445 ***	0.166 ***	0.578 ***	−0.058
	(0.075)	(0.029)	(0.083)	(0.071)
N	1938	1938	1938	1938
AR(1) (*p*-values)	0.000	0.000	0.000	0.000
AR(2) (*p*-values)	0.746	0.261	0.994	0.262
Hansen (*p*-values)	0.261	0.432	0.537	0.522

Notes: The value in brackets are the standard errors. About them: *, **, *** respectively indicate that the estimated parameters pass the statistical significance test at 10%, 5%, and 1%.

**Table 5:** Regression results of mechanism analysis for foreign direct investment.

Variables	1	2	3
	FDI	CE	CEE
L.FDI	0.815 ***		
	(0.013)		
L.CE		0.946 ***	
		(0.016)	
L.CEE			0.709 ***
			(0.022)
ER	0.021 ***	−0.011 ***	0.005 ***
	(0.006)	(0.003)	(0.001)
FDI		−0.018 **	0.011 ***
		(0.006)	(0.001)
Controls	Yes	Yes	Yes
Constant	0.819 ***	0.551 ***	0.072
	(0.149)	(0.089)	(0.072)
N	1938	1938	1938
AR(1) (*p*-values)	0.000	0.000	0.000
AR(2) (*p*-values)	0.429	0.953	0.274
Hansen (*p*-values)	0.337	0.625	0.476

Notes: The value in brackets are the standard errors. About them: **, *** respectively indicate that the estimated parameters pass the statistical significance test at 5%, and 1%.

**Table 6:** Regression results of mechanism analysis for green technology innovation.

Variables	1	2	3
	GTI	CE	CEE
L.GTI	0.248 ***		
	(0.008)		
L.CE		0.959 ***	
		(0.013)	
L.CEE			0.717 ***
			(0.045)
ER	0.057 ***	−0.012 ***	0.006 ***
	(0.009)	(0.003)	(0.02)
GTI		−0.018 ***	0.008 **
		(0.003)	(0.003)
Controls	Yes	Yes	Yes
Constant	−0.779 **	0.350 ***	0.017
	(0.329)	(0.094)	(0.089)
N	1938	1938	1938
AR(1) (*p*-values)	0.000	0.000	0.000
AR(2) (*p*-values)	0.424	0.713	0.294
Hansen (*p*-values)	0.743	0.713	0.376

Notes: The value in brackets are the standard errors. About them: **, *** respectively indicate that the estimated parameters pass the statistical significance test at 5%, and 1%.

**Table 7:** Regression results of mechanism analysis for industrial structure upgrading.

Variables	1	2	3
	ISU	CE	CEE
L.ISU	0.837 ***		
	(0.023)		
L.CE		0.944 ***	
		(0.015)	
L.CEE			0.739 ***
			(0.028)
ER	0.006 ***	−0.014 ***	0.006 ***
	(0.011)	(0.003)	(0.001)
ISU		−0.036 **	0.008
		(0.016)	(0.007)
Controls	Yes	Yes	Yes
Constant	0.907 ***	0.638 ***	−0.018 ***
	(0.119)	(0.084)	(0.056)
N	1938	1938	1938
AR(1) (*p*-values)	0.000	0.000	0.000
AR(2) (*p*-values)	0.305	0.739	0.262
Hansen (*p*-values)	0.373	0.618	0.530

Notes: The value in brackets are the standard errors. About them: **, *** respectively indicate that the estimated parameters pass the statistical significance test at 5%, and 1%.

**Table 8:** Robustness test.

Variables	Winsorization		Delete Strictly Regulated Areas		Add Explanatory Variable Lag Term		Replace ER	
	1	2	3	4	5	6	7	8
	CE	CEE	CE	CEE	CE	CEE	CE	CEE
L.CE	0.968 ***		0.949 ***		0.947 ***		0.949 ***	
	(0.009)		(0.014)		(0.014)		(0.013)	
L.CEE		0.676 ***		0.596 ***		0.738 ***		0.758 ***
		(0.030)		(0.029)		(0.082)		(0.032)
ER	−0.014 ***	0.005 ***	−0.014 ***	0.005 ***	−0.014 ***	0.054 *		
	(0.002)	(0.001)	(0.003)	(0.001)	(0.003)	(0.003)		
L.ER					−0.001	0.017		
					(0.003)	(0.003)		
R-ER							−0.069 ***	0.046 ***
							(0.029)	(0.012)
Controls	Yes	Yes	Yes	Yes	Yes	Yes	Yes	Yes
Constant	0.568 ***	0.029	0.562 ***	0.109 *	0.573 ***	−0.019	0.735 ***	0.162 **
	(0.062)	(0.106)	(0.082)	(0.060)	(0.089)	(0.164)	(0.064)	(0.068)
N	1823	1823	1751	1751	1938	1938	1938	1938
AR(1) (*p*-values)	0.000	0.000	0.000	0.000	0.000	0.000	0.000	0.000
AR(2) (*p*-values)	0.558	0.109	0.901	0.450	0.985	0.278	0.674	0.284
Hansen (*p*-values)	0.544	0.276	0.805	0.848	0.511	0.483	0.514	0.375

Notes: The value in brackets are the standard errors. About them: *, **, *** respectively indicate that the estimated parameters pass the statistical significance test at 10%, 5%, and 1%.

**Table 9:** Regression results at different types of RBCs.

Variables	Maturity Type		Growth Type		Recession Type		Regeneration Type	
	1	2	3	4	5	6	7	8
	CE	CEE	CE	CEE	CE	CEE	CE	CEE
L.CE	0.855 ***		0.859 *		0.904 ***		0.845 ***	
	(0.077)		(0.155)		(0.067)		(0.059)	
L.CEE		0.401 ***		0.304 *		0.374 ***		0.263
		(0.021)		(0.142)		(0.045)		(0.250)
ER	−0.032 **	0.007 ***	−0.076 *	0.008	−0.019	−0.001	−0.022 *	0.032 *
	(0.011)	(0.001)	(0.037)	(0.013)	(0.017)	(0.004)	(0.012)	(0.017)
Controls	Yes	Yes	Yes	Yes	Yes	Yes	Yes	Yes
Constant	0.951 **	0.226 ***	0.623	0.634 **	0.345	0.041	0.483 *	0.458
	(0.276)	(0.022)	(0.847)	(0.289)	(0.465)	(0.145)	(0.250)	(1.033)
N	1054	1054	238	238	391	391	255	255
AR(1) (*p*-values)	0.000	0.000	0.036	0.041	0.001	0.001	0.013	0.071
AR(2) (*p*-values)	0.870	0.723	0.148	0.915	0.980	0.860	0.585	0.112
Hansen (*p*-values)	0.381	0.540	0.888	0.976	0.148	0.496	0.959	0.601

Notes: The value in brackets are the standard errors. About them: *, **, *** respectively indicate that the estimated parameters pass the statistical significance test at 10%, 5%, and 1%.

**Table 10:** Regression results at different regions.

Variables	Northeast Region		Non-Northeast Region	
	1	2	3	4
	CE	CEE	CE	CEE
L.CE	0.964 ***		0.651 ***	
	(0.013)		(0.155)	
L.CEE		0.455 ***		0.231 *
		(0.024)		(0.117)
ER	−0.014 ***	0.005 **	−0.012	−0.004
	(0.004)	(0.002)	(0.007)	(0.003)
Controls	Yes	Yes	Yes	Yes
Constant	0.541 ***	0.294 **	1.734 **	−0.065
	(0.081)	(0.052)	(0.720)	(0.303)
N	1530	1530	323	323
AR(1) (*p*-values)	0.000	0.000	0.013	0.008
AR(2) (*p*-values)	0.611	0.908	0.467	0.754
Hansen (*p*-values)	0.926	0.473	0.953	0.998

Notes: The value in brackets are the standard errors. About them: *, **, *** respectively indicate that the estimated parameters pass the statistical significance test at 10%, 5%, and 1%.

## Data Availability

The data used to support the findings of this study are available from the corresponding author upon request.
